# Supplementary material for: The Systems Biology Research Tool: evolvable open-source software
Source: BMC Syst Biol. 2008 Jun 29;2:55. doi: 10.1186/1752-0509-2-55 (PMC2446383; doi:10.1186/1752-0509-2-55)
Supplement: Additional file 1 — SBRT Archive. An archive of the current version of the Systems Biology Research Tool. [file 1752-0509-2-55-S1.zip › sbrt-1.4.0/doc/developers_guide/api/sbrt/kernel/fba/ExchangeRxn.html]

ExchangeRxn


|  |  |  |
| --- | --- | --- |
| **Overview** \| **Package** \| **Class** \| **Use** \| **Tree** \| **Deprecated** \| **Index** \| **Help** | |  |
| **PREV CLASS**   **NEXT CLASS** | **FRAMES**    **NO FRAMES**     **All Classes** |  |
| SUMMARY: NESTED \| FIELD \| CONSTR \| METHOD | DETAIL: FIELD \| CONSTR \| METHOD |  |


---


## sbrt.kernel.fba Class ExchangeRxn

```
java.lang.Object
  sbrt.kernel.fba.ExchangeRxn
```

**All Implemented Interfaces:**: ChemRxn

**Direct Known Subclasses:**: SinkRxn, SourceRxn

---

``` public abstract class ExchangeRxn extends java.lang.Object implements ChemRxn ```

This class is used to represent exchange reactions in Flux Balance Analysis.
An exchange reaction has a name and is associated with a single chemical species.

Instances of this class are immutable.

**Author:**
:   This class was written and documented by Jeremiah Wright
    while in the Wagner lab.

---

**Constructor Summary**

`ExchangeRxn(java.lang.String chemSpecies)`
  
          Constructs a new exchange reaction for the provided chemical species.


| **Method Summary** | |
| --- | --- |
| `boolean` | `equals(java.lang.Object obj)`             Indicates if the provided object is equal to this exchange reaction. |
| `LinearComb<java.lang.String>` | `getProducts()`             Returns an empty linear combination. |
| `LinearComb<java.lang.String>` | `getReactants()`             Returns an empty linear combination. |
| `java.lang.String` | `getSpecies()`             Returns the chemical species associated with this exchange reaction. |
| `int` | `hashCode()`             Returns a content-based hash code for this exchange reaction. |

**Methods inherited from class java.lang.Object**

`clone, finalize, getClass, notify, notifyAll, toString, wait, wait, wait`

**Methods inherited from interface sbrt.kernel.fba.ChemRxn**

`isEquivalent, isReverse, toString`

**Constructor Detail**

### ExchangeRxn

```
public ExchangeRxn(java.lang.String chemSpecies)
```

:   Constructs a new exchange reaction for the provided chemical species.

    **Parameters:**: `chemSpecies` - the chemical species associated with this exchange reaction.


**Method Detail**

### getSpecies

```
public final java.lang.String getSpecies()
```

:   Returns the chemical species associated with this exchange reaction.

    :   **Returns:**: the chemical species associated with this exchange reaction.

---


### getReactants

```
public LinearComb<java.lang.String> getReactants()
```

:   Returns an empty linear combination.

    :   **Specified by:**: `getReactants` in interface `ChemRxn`
    :   **Returns:**: an empty linear combination.

---


### getProducts

```
public LinearComb<java.lang.String> getProducts()
```

:   Returns an empty linear combination.

    :   **Specified by:**: `getProducts` in interface `ChemRxn`
    :   **Returns:**: an empty linear combination.

---


### equals

```
public boolean equals(java.lang.Object obj)
```

:   Indicates if the provided object is equal to this exchange reaction.
    For two exchange reactions `r1` and `r2` to be equal,
    `r1.isEquivalent(r2)` and
    `r1.getName().equals(r2.getName())` must both return
    `true`.

    :   **Specified by:**: `equals` in interface `ChemRxn` **Overrides:**: `equals` in class `java.lang.Object`
    :   **Parameters:**: `obj` - the reference object with which to compare. **Returns:**: `true` if this exchange reaction is equal to the provided object; `false` otherwise. **See Also:**: `ChemRxn.isEquivalent(ChemRxn)`

---


### hashCode

```
public int hashCode()
```

:   Returns a content-based hash code for this exchange reaction.

    :   **Specified by:**: `hashCode` in interface `ChemRxn` **Overrides:**: `hashCode` in class `java.lang.Object`
    :   **Returns:**: a content-based hash code for this exchange reaction.


---


|  |  |  |
| --- | --- | --- |
| **Overview** \| **Package** \| **Class** \| **Use** \| **Tree** \| **Deprecated** \| **Index** \| **Help** | |  |
| **PREV CLASS**   **NEXT CLASS** | **FRAMES**    **NO FRAMES**     **All Classes** |  |
| SUMMARY: NESTED \| FIELD \| CONSTR \| METHOD | DETAIL: FIELD \| CONSTR \| METHOD |  |


---
